# Application of Microneedles for High-Molecular-Weight Dextran Penetration Across the Buccal Mucosa

**DOI:** 10.3390/ph18020158

**Published:** 2025-01-25

**Authors:** Adriana Fantini, Andrea Delledonne, Luca Casula, Sara Nicoli, Silvia Pescina, Maria Cristina Cardia, Francesco Lai, Cristina Sissa, Patrizia Santi, Cristina Padula

**Affiliations:** 1 ADDRes Lab, Department of Food and Drug, University of Parma, Parco Area delle Scienze 27/a, 43124 Parma, Italy; fantiniadriana@gmail.com (A.F.); sara.nicoli@unipr.it (S.N.); silvia.pescina@unipr.it (S.P.); patrizia.santi@unipr.it (P.S.); 2 Department of Chemistry, Life Science and Environmental Sustainability, University of Parma, Parco Area delle Scienze 17/a, 43124 Parma, Italy; andrea.delledonne@unipr.it (A.D.); cristina.sissa@unipr.it (C.S.); 3 Department of Life and Environmental Sciences, University of Cagliari, Via Ospedale 72, 09124 Cagliari, Italy; luca.casula@unica.it (L.C.); cardiamr@unica.it (M.C.C.); frlai@unica.it (F.L.)

**Keywords:** microneedles, dextran, permeation, buccal mucosa

## Abstract

**Objectives:** This work aimed at investigating the effect of different microneedle-based strategies on the permeation of high-molecular-weight model molecules (fluorescently labeled dextrans (FDs), 70 and 150 kDa) across the buccal mucosa. **Methods:** Two different approaches were evaluated: (1) stainless steel microneedles (MNs) of 500 µm height used for tissue pre-treatment; and (2) soluble microneedles of different lengths (150, 500, and 800 µm), made of polyvinylpyrrolidone and FDs, prepared using the solvent casting technique. Porcine esophageal epithelium was used as a model for the buccal mucosa. **Results:** The application of soluble MNs promoted high-molecular-weight dextran transport across pig esophageal epithelium. The transport was proportional to MN length, with a minimum of 500 µm, regardless of the molecular weight of the FDs. The use of solid MNs of the same length to pre-treat the tissue, followed by the application of a solution of the permeant, did not produce the same effect in terms of onset of permeation, which was found to be much slower. **Conclusions:** The results obtained show that by applying soluble MNs of appropriate length (500 and 800 µm), the transport of high-molecular-weight dextrans (70 and 150 kDa) across and into the mucosal tissue occurs very rapidly. The multiphoton microscopy analysis confirmed the presence of holes in the tissue and the presence of fluorescein-labeled dextrans.

## 1. Introduction

Macromolecules, including proteins and peptides, antibodies, oligonucleotides, and polysaccharides, represent the most promising new chemical entity class, because of their advantages in terms of tolerability, specificity, efficacy, and potency [1]. In fact, of the new drugs approved in 2023 by the Food and Drug Administration, only 50% were small molecules, which a few years ago accounted for more than 80% of all marked drugs [2]. Of the 55 new drugs approved, 26 were macromolecules, specifically 12 monoclonal antibodies, 5 proteins/enzymes, 5 peptides, and 4 oligonucleotides. In most of the cases, owing to their physicochemical properties, macromolecule administration requires the parenteral route; the drawbacks of this method (poor patient compliance, cost, risk of infections, etc.) stimulated researchers to find alternative administration routes [3]. Oromucosal administration, i.e., administration of the drug through the buccal or sublingual mucosa, has recently received a certain interest, also for macromolecular drugs. Despite the many advantages (accessibility, low enzymatic activity, avoidance of first pass effect, etc.), the mucosal delivery of high-molecular-weight drugs is still challenging due to their large molecular weight, hydrophilic nature, and poor permeability across epithelia. Different strategies to increase macromolecule penetration have been proposed, such as chemical penetration enhancers, enzyme inhibitors, pro-drugs, mucoadhesive materials, and microneedles [1]. Chemical penetration enhancers (surfactants, bile salts, fatty acids, Azone, ethanol, etc.) have received a certain attention for their ability to increase skin and mucosa permeation [4]; however, their use is associated with possible irritation, membrane damage, and toxicity [5].

In a previous work [6], we studied the effect of several penetration enhancers (fatty acids and bile salts) on fluorescently labeled dextran (m.w. 4000 Da) penetration; these preliminary data were then used to evaluate the buccal mucosa penetration of high-molecular-weight dextrans (up to 150 kDa) [7]. The results obtained represent the proof of concept of the possibility to promote the mucosal penetration of high-molecular-weight dextrans (70 and 150 kDa) using caprylic acid in pre-treatment or sodium taurocholate in co-administration. The use of chemical enhancers is not free from problems related to the above-mentioned possible toxic effects and to the need of a complex formulation and/or a specific application protocol, in particular in the case of pre-treatment [7].

Microneedles (MNs) are micron-sized needles, on a solid support, with heights ranging between 25 and 2000 µm, that can pierce biological tissues and create microchannels [8]. The concept of MNs was first presented in a patent in 1971 [9]; since then, a wide variety of MNs have been developed in terms of type, material, and shape, mostly for skin application. MNs are conventionally applied to the skin with a patch or a roller or an applicator and, at present, there are five main types of microneedles: solid, dissolving, hollow, coated, and hydrogel-forming [10]. Solid MNs deliver drugs by creating microchannels in the stratum corneum and promoting the release of drug molecules to and across the tissue [11]. Dissolving MNs, made of soluble excipients, dissolve after insertion into the tissue; they do not require a removal step, making them more patient-friendly. Dissolving MNs can deliver a broad range of drugs and therapeutic high-molecular-weight molecules, following the needle’s dissolution inside the skin tissue [12]. Several geometrical parameters can affect skin penetration of solid MNs, such as shape [13], needle length [14], tip and base width/diameter [15], and pitch [16].

The application of MNs to the buccal mucosa is more recent and less characterized, compared to their application to the skin. In a recent review [17], it was concluded that MNs have the potential to produce a better treatment outcome with reduced side effects. MN application has also been explored for vaccine [18] and peptide (human insulin and human growth hormone) [19] administration.

This work aimed at investigating the effect of different microneedle-based strategies on high-molecular-weight model molecule permeation through porcine esophageal epithelium. Fluorescently labeled dextrans (FDs) with 70 kDa and 150 kDa molecular weight were used as a model for therapeutic high-molecular-weight compounds, because they have been shown not to cross the buccal epithelium in passive conditions [7]. Two different approaches were evaluated: (1) stainless steel MNs of 500 µm height used for tissue pre-treatment before FD solution application; and (2) soluble microneedles, containing PVP as a soluble and biocompatible polymer and FDs, prepared using the solvent casting technique. These MNs had a pyramid shape, and three different lengths were tested, 150, 500, and 800 µm.

## 2. Results and Discussion

### 2.1. Soluble MN Characterization

**Figure 1:**
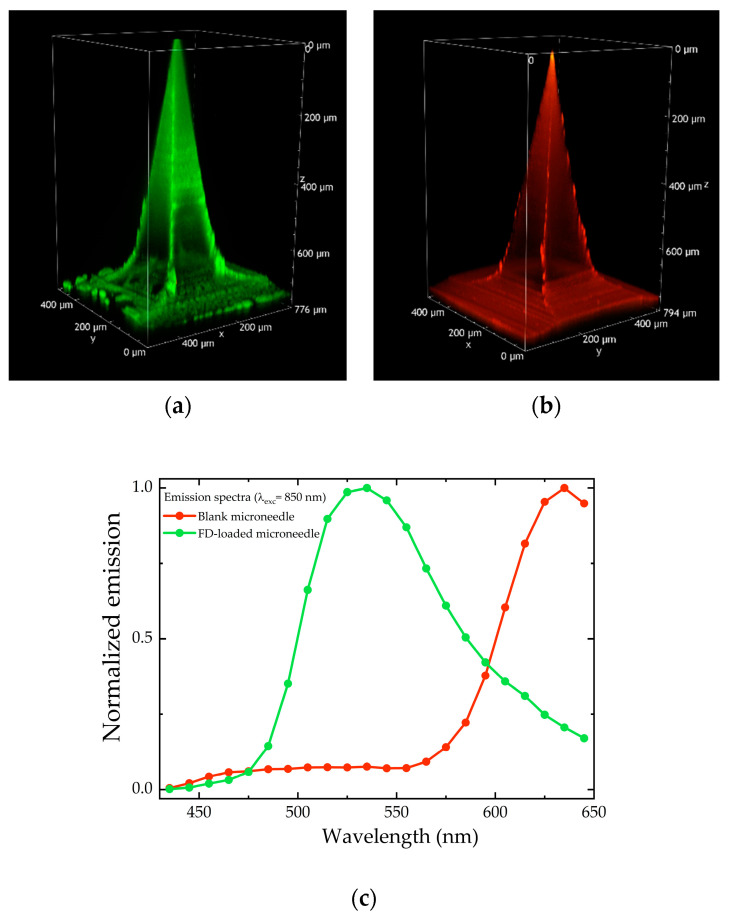
Volume rendering of 800 µm microneedles, FD-70-loaded (**a**) and blank (**b**), reconstructed from the Z-stacks (total depth: 776 and 794 µm, respectively; Z-step: 2 µm), acquired with an excitation wavelength of 850 nm. The gain and laser power employed to acquire the images for panel (**b**) were higher compared to the experimental settings for panel (**a**). Normalized emission spectra of blank and FD-loaded microneedles (**c**).

Polyvinyl pyrrolidone was chosen as a dissolvable polymer, because it has been shown to produce MNs of sufficient strength to penetrate the skin, is biocompatible, and exhibits low toxicity [14]. Both “blank” and FD-loaded microneedles were studied, collecting 3D images (Z-stacks) and emission spectra, using two-photon microscopy. Figure 1 shows a representative example of 800 µm MNs loaded with FD-70 (panel a) and blank (panel b).

The shape and size of the microneedles were in line with the values expected from mold size: pyramidal shape, approximately 800 µm in height, and 200 × 200 µm base. The emission spectra shown in Figure 1 (panel c) suggest that the green emission of the FD-70-loaded MN comes from the fluorescein present in dextran, while the red fluorescence detected in the “blank” MN probably comes from the polyvinylpyrrolidone (PVP) scaffold itself (or from some impurities).

MN arrays were also characterized in terms of rate of dissolution, by measuring the amount of FD-70 released over time from single arrays placed in 5 mL of pH 7.4 buffer solution. The results show that, when placed in aqueous medium, MNs dissolved in less than 4 min, in agreement with the reported quick dissolution of the polymer [14]. The average amount of FD-70 loaded was approx. 800 µg per array.

### 2.2. Permeation Studies

In this work, microneedle patches with different needle lengths and containing different-molecular-weight fluorescent dextrans were tested in vitro using pig esophageal epithelium as the barrier.

#### 2.2.1. Stainless Steel MNs vs. Soluble MNs

**Figure 2:**
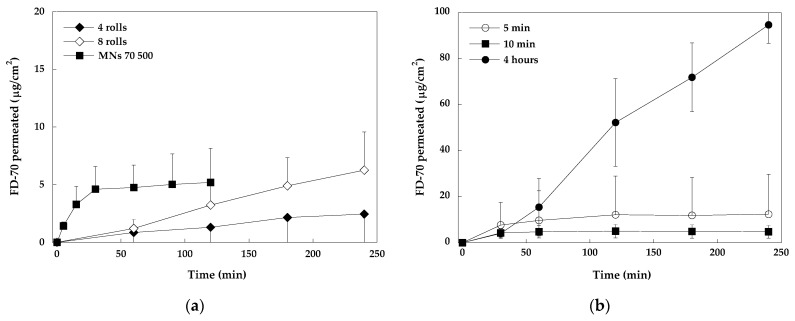
(**a**) Permeation curves of FD-70 with dermaroller (500 µm) pre-treatment (4 or 8 rolls), followed by the application of a 2 mg/mL solution of FD-70; (**b**) permeation curves of FD-70 after the application of soluble MN (500 µm) patches for 5 min., 10 min, and 2 h. Mean values ± SD (for the sake of clarity, SD is sometimes represented only in one direction).

At first, stainless steel MNs were evaluated. In analogy with the data published on the skin [20], the dermaroller cylinder (MNs of 500 µm in length) was rolled on the mucosa surface four or eight times, and then a 2 mg/mL FD-70 solution was applied. The results obtained, reported in Figure 2 (panel a), demonstrated that MNs can pierce the buccal tissue (the corresponding passive experiments were zero), although probably due to the elasticity of the tissue, the micropores created did not allow the important penetration of FD-70. Additionally, the permeation kinetic was very slow, the data are characterized by a very high variability, and no statistical difference between four and eight passes is present. Research data on MN application to the skin, using a dermaroller cylinder, shows that micropores created in the skin increase at each pass of the roller [20], but again, the elasticity of the epithelial tissue may reduce this effect. In fact, the elastic modulus of the skin is reported to be approx. 19.6 MPa, whereas the buccal mucosa shows a value of approx. 8 MPa [21] and the esophagus of approx. 2 MPa [22], demonstrating a higher elasticity.

Considering these results, and the difficulties in the practical application of this approach to the mucosa, the use of this system was discarded and only soluble MNs were further investigated.

Preliminary experiments with soluble MNs were performed to find the more appropriate conditions for in vitro experiments (Figure 2, panel b). First of all, the application time was determined: the length of time during which the array is applied to the mucosa should be long enough to guarantee MN dissolution, but not too long to produce patient discomfort. MNs (500 µm length, loaded with FD-70) were applied to the mucosa for 5 min, 10 min, and 2 h (in all cases, FD permeation was followed up to 4 h). The obtained results show that 5 min is too short to allow for the complete dissolution of MNs inside the tissue: when the array was removed, it was possible to observe that some MNs were still undissolved and, at the end of the experiment, the variability obtained was very high. As observed also by other authors [23], the limited amount of water inside the tissue reduced the dissolution rate of the polymer and dextran, if compared with the conditions of the dissolution experiment, in which MNs dissolved in 4 min (see Section 2.1. Soluble MN Characterization). When MNs were applied to the tissue for the whole length of the experiment (as was done in the case of skin application), a large amount of dextrans penetrated the skin; however, this condition is uncomfortable for the patient. An application time of 10 min was found to be adequate to guarantee MN dissolution and this application time can be considered acceptable for the patient.

Considering these results, in all subsequent experiments, MN patches were applied to the mucosa for 10 min and then removed, whereas the release of dextrans across the tissue was monitored for up to 2 h, because after 2 h the release profile flattened, indicating that the available FD had already penetrated across the tissue.

#### 2.2.2. In Vitro Evaluation of Dissolving Microneedle Arrays

**Figure 3:**
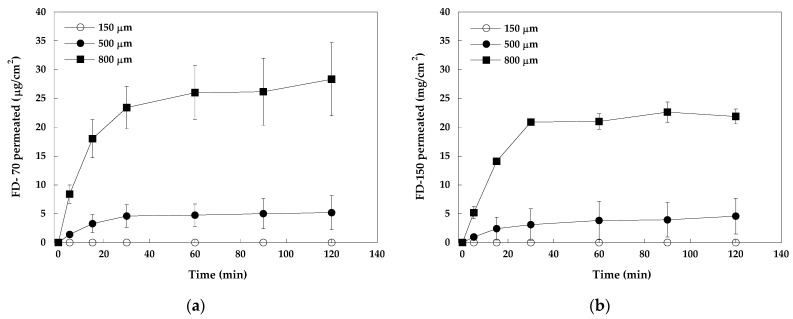
(**a**) Permeation curves of FD-70 after the application of soluble MN patches of different lengths for 10 min. (**b**) Permeation curves of FD-150 after the application of soluble MN patches of different lengths for 10 min. Mean values ± SD.

Figure 3 shows the permeation profiles of FD-70 (panel a) and FD-150 (panel b) across pig esophageal epithelium after the application of patches with three different needle lengths, namely, 150, 500, and 800 µm, for 10 min. High-molecular-weight dextrans permeated the mucosa very quickly, within a few minutes, and the extent was highly dependent on the length of the needles. After the application of 150 μm MNs, no dextran permeation was observed; probably the MNs were too short to cross the membrane, which had a thickness of approx. 400 µm (consistent with the data in the literature, reporting an average thickness of pig epithelium of 340–490 µm [24]). Additionally, it might be necessary to have a minimum needle length to overcome the elasticity of the mucosa, as has been reported for the skin [25].

When 500 µm MNs were used, both dextrans, FD-70 and FD-150, penetrated the tissue quite quickly and to the same extent. The same was observed with 800 µm, although the total amount permeated was higher (not significantly different for the two dextrans; *p* > 0.05; *t*-test). Considering the effect of needle length on the permeation of the individual dextrans, in both cases, the transport was significantly higher for all time points (*p* < 0.005 for FD-70 and *p* < 0.05 for FD-150; *t*-test). Longer needles (800 µm) can probably cross the whole tissue, whereas shorter ones (500 µm) cannot do the same, although they are nominally longer than tissue thickness (400 µm). The literature reports that when using soluble MNs, the penetration depth in the skin is considerably lower than the MNs’ length [26]. This difference has also been observed in the buccal mucosa: using 500 µm soluble MNs, the real penetration depth, as observed using confocal microscopy, was approx. 300 µm [23].

The results obtained also show that the molecular weight of the permeant has a limited effect; in fact, FD-150 permeated in slightly smaller amounts compared to FD-70, but the differences were not statistically significant (*p* > 0.05; *t*-test). This result is somewhat surprising, although it has already been observed. In fact, no difference in the permeability of FD-70 and FD-150 was found by (i) Matsukawa et al. across rat alveolar epithelium (the hypothesized mechanism is pinocytosis) [27], (ii) Fantini et al. across pig esophageal epithelium using chemical penetration enhancers (high-molecular-weight dextrans permeate through the opening of tight junctions [7]), and Ambati et al. across rabbit sclera (the hypothesized dextrans are transported by porous diffusion through a fiber matrix [28]), or (iii) Hutton et al. across the skin from hydrogel-forming MNs [29]. In the present work, the absence of differences is probably due to the mechanism of dissolvable MNs: they pierce the membrane, creating holes, and then dissolve, releasing the active molecules into and/or across the tissue (depending on their length).

Comparing these results with those obtained using chemical penetration enhancers [7], it is evident that MN application allows a very quick onset of action, whereas non-invasive techniques require several hours.

#### 2.2.3. Two-Photon Microscopy Analysis

Mucosa samples treated with FD-70-loaded MNs were imaged by two-photon microscopy, to demonstrate the presence of fluorescent dextran released from soluble MNs in the tissue. To this end, images detecting the emission from FDs in the green channel (506–593 nm) and the tissue autofluorescence in the red one (604–679 nm), upon excitation at 1030 nm, were acquired. The detector gain of the red channel was set at a value higher than the green one, to enhance the contrast between fluorescein and tissue emission. The results obtained are as follows:

- 150 µm MNs loaded with FD-70

No significant information was obtained from this type of MNs. Possibly, the microneedles were too short to penetrate the tissue, in agreement with the permeation data (see Section 2.2.2. In Vitro Evaluation of Dissolving Microneedle Arrays). As mentioned before, the literature reports that the average thickness of the pig epithelium is approx. 400 µm [24,30], and this agrees with our microscopy images (see below).

- 500 µm MNs loaded with FD-70

**Figure 4:**
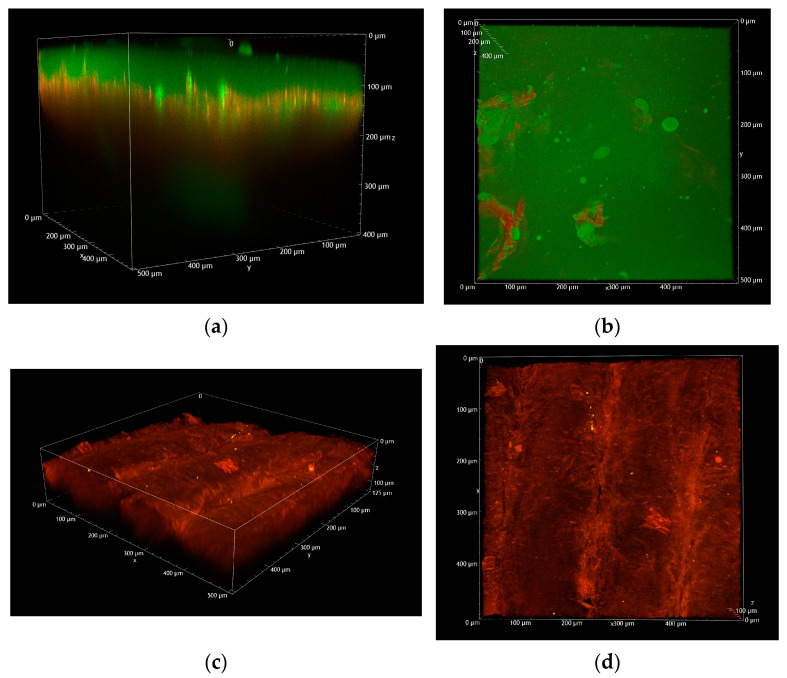
(**a**) Volume rendering overview of a fluorescent 500 µm MN applied to porcine esophagus reconstructed from the Z-stack (total depth: 400 µm; Z-step: 2 µm); (**b**) XY view; (**c**) volume rendering overview of untreated porcine esophagus reconstructed from the Z-stack (total depth: 125 µm; Z-step: 2 µm); (**d**) XY view. All the images were acquired with an excitation wavelength of 1030 nm and the same detector gains.

Figure 4 shows volume renderings reconstructed from Z-stacks, displaying an overlay of the green and red channels. Panel (a) displays the 3D overview of a single FD-70-loaded 500 µm MN applied to pig esophageal epithelium, while panel (b) shows the corresponding XY view. For comparison purposes, panels (c) and (d) display the equivalent images of an untreated tissue, acquired under identical experimental conditions.

To minimize polymer dissolution, the images of tissue treated with MNs, taken just after array application, were acquired without using water to dip the objective. This procedure negatively affects the quality of the images both in terms of resolution and brightness compared to measurements where water is used to establish contact between the objective and the sample. Despite this precaution, the water contained in the tissue and air humidity led to the partial or complete dissolution of microneedles.

The white arrow in Figure 4 (panel a) indicates the presence of a partially dissolved MN inside the tissue, at a depth of approx. 300 µm from the surface. This is in agreement with data from the literature, showing that using 500 µm soluble MNs, the real penetration depth, as observed using confocal microscopy, was approx. 300 µm [23]. The surface of the sample is covered by the green signal from fluorescein emission, originating from the completely dissolved PVP array (panel b).

**Figure 5:**
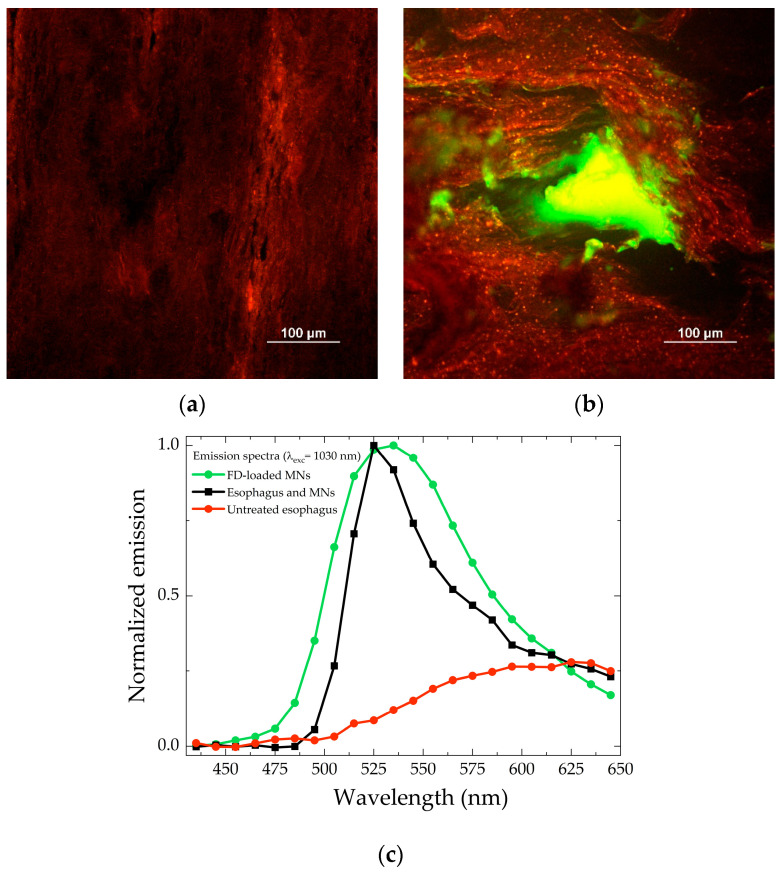
(**a**) XY image of untreated porcine esophagus. (**b**) XY image of a 500 µm MN applied to porcine esophagus. Both images were acquired between 50 and 100 µm below the tissue surface with an excitation wavelength of 1030 nm and the same detector gains. (**c**) Comparison between normalized emission spectra acquired in correspondence to panel a (red line) and panel b (black line) focal planes and the emission of the FD-loaded microneedle (green line). The autofluorescence of the tissue (red line) has been arbitrarily normalized to show its contribution to the spectrum of panel b.

Figure 5 shows the image obtained by collecting the autofluorescence of the untreated tissue (panel a) and the image of a “hole” in the tissue caused by a fluorescent MN (panel b). Emission spectra collected in correspondence to panel a (red line) and panel b (black line) focal planes and the emission of the FD-loaded microneedle (green line) are reported in Figure 5 (panel c): the emission spectrum collected from panel b clearly shows both the presence of fluorescein (peak at 525 nm) and the autofluorescence of the tissue (broad band at 500–650 nm).

- 800 µm microneedles loaded with FD-70

**Figure 6:**
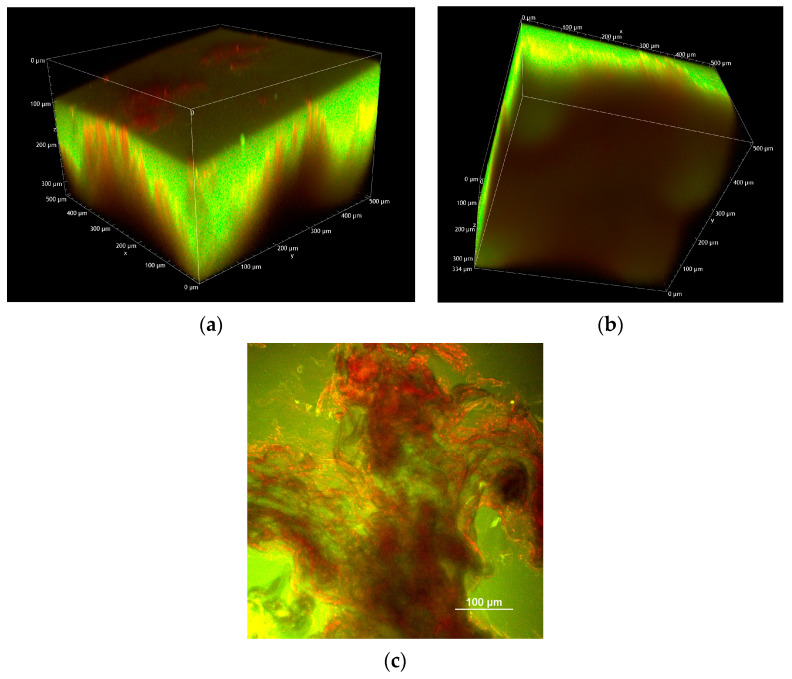
(**a**,**b**) Volume rendering overview of four FD-70-loaded 800 µm MNs applied to porcine esophagus, reconstructed from the Z-stack (total depth: 334 µm; Z-step: 2 µm). (**c**) XY image acquired about 150 µm below the sample surface. All the images were acquired with an excitation wavelength of 1030 nm.

In Figure 6, it is possible to observe four different 800 µm MNs within the esophageal tissue.

**Figure 7:**
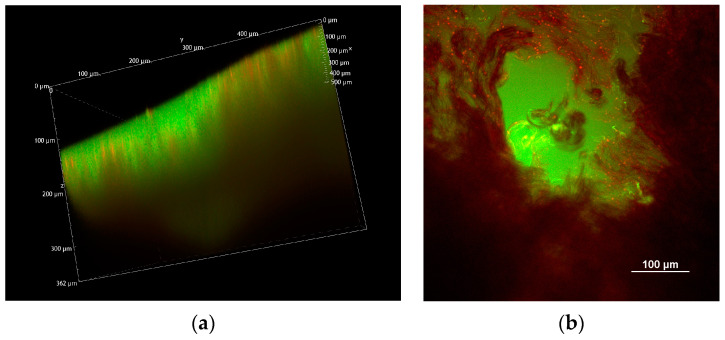
(**a**) Volume rendering overview of a fluorescent 800 µm MN applied to porcine esophagus, reconstructed from the Z-stack (total depth: 362 µm; Z-step: 2 µm). (**b**) XY image acquired about 150 µm below the sample surface. All images were acquired with an excitation wavelength of 1030 nm.

In Figure 7, a volume rendering of a different region of the same sample is shown, with a single MN penetrating the tissue.

## 3. Materials and Methods

### 3.1. Materials

Fluorescein isothiocyanate labeled dextrans of 70 (FD-70) and 150 (FD-150) KDa were purchased from Sigma-Aldrich (St. Louis, MO, USA); Polyvinylpirrolidone 10,000 m.w. (PVP) was from Sigma-Aldrich (St. Louis, MO, USA). All other reagents were of analytical grade.

MPatch™ Microneedle Templates (10 × 10 array; pyramid, base = 200 × 200 μm; H = 150, 500, and 800 μm; pitch 500 µm) were obtained from Micropoint Technologies Pte Ltd., Singapore.

The microneedle roller used (Derma Roller System, TinkSky^®^, Prolinx GmbH, D) possesses 60 circular arrays of 9 needles (500 µm long; pitch 1000 µm; 540 needles in total) in a cylindrical assembly.

### 3.2. Methods

#### 3.2.1. Analytical Method

The concentration of FDs in the samples was determined using a Spark multimode microplate reader (TECAN, Mannendorf, CH), as reported in Fantini et al. [7]. The excitation and emission λ were 490 and 520 nm, respectively. A standard curve was developed by plotting different concentrations of FDs in PBS (ng/mL) versus their fluorescence values. The analytical method was validated for specificity, linearity, and limit of quantification for each dextran.

#### 3.2.2. Preparation of PVP Dissolving Microneedles

The preparation of soluble microneedles was performed with a slight variation in the method already reported [31]. Firstly, 1 mL of water solution of FD 70 or FD 150 (25 mg/mL) was added to 0.8 g of PVP. Then, 60 µL of the obtained dispersion was dropped in silicon templates (150, 500, and 800 µm tip height; 100 microneedles per cm^2^) and centrifuged at 4000 rpm for 15 min to allow the penetration and homogeneous distribution of the suspension in the MN tips and to remove bubbles. After 24 h of drying at room temperature, the template was filled several times with a dispersion of PVP (0.8 g/mL) until a solid and flat base was created for the microneedle patch. Final drying was performed at room temperature in a glass desiccator jar for 48–72 h. The microneedle patches obtained were observed with two-photon microscopy, after gentle extraction from the template and application to the porcine esophagus; they were also used to perform ex vivo permeation experiments.

#### 3.2.3. Permeation Studies

Ex vivo permeation studies were conducted across porcine esophageal epithelium. Pig esophagi (Large White or Landrace pigs; age: 11–12 months; weight: 145–190 kg) were obtained from a local slaughterhouse within 2 h from animal sacrifice. The esophageal mucosa was detached from the outer muscle layer with a scalpel and the epithelium was peeled off from the connective tissue after immersion in distilled water at 60 °C for 60 s [24]. The samples obtained were frozen until use, which occurred within 3 months.

##### In Vitro Evaluation of Dissolving Microneedle Patches

A microneedle patch was applied to porcine tissue, by pressing with the operator’s thumb. After 10 min, the patch was removed and the mucosa, supported by a regenerated cellulose filter (0.45 µm pore size), was mounted between the donor and receptor compartments of Franz’s type diffusion cells (DISA, Milan, I) of 0.6 cm^2^ permeation area. The receptor compartment (volume 4 mL) was filled with pH 7.4 PBS, and FITC-dextran permeation was monitored up to 2 h, taking 300 µL samples at predeterminate time intervals and replacing them with the same amount of fresh PBS. The collected samples were analyzed as previously indicated; samples from 800 and 500 µm MN patches were diluted 1:5 before analysis, to keep them inside the linearity range [7], whereas samples from 150 µm MN patches were analyzed without dilution.

All experiments were performed at 37 °C.

##### In Vitro Evaluation of Stainless Steel Microneedles

Some samples of mucosa were treated with solid stainless steel MNs of 500 µm length (70 MNs/cm^2^) mounted on a cylindrical roller. The roller was passed on the tissue 4 or 8 times, and then the treated tissue was mounted, using a regenerated cellulose filter, in Franz’s type diffusion cells (0.6 cm^2^). The donor compartment was filled with 400 µL of FD solution (2 mg/mL) in pH 7.4 PBS, and the experiment was carried out as indicated before. In this case, the experiment lasted 7 h. All experiments were performed at 37 °C.

#### 3.2.4. Two-Photon Microscopy

Specimens were analyzed with a Two-Photon Microscope Nikon A1R MP+ Upright (Nikon, Tokyo, J) equipped with a femtosecond pulsed laser Coherent Chameleon Discovery (~100 fs pulse duration with 80 MHz repetition rate; tunable wavelength output 660–1320 nm). A 25× water dipping objective with numerical aperture 1.1 and 2 mm working distance was employed to focus the excitation beam and to collect the two-photon excited fluorescence (TPEF) signal. The TPEF signal was directed by a dichroic mirror to a series of two non-descanned detectors (high-sensitivity GaAsP photomultiplier tubes), allowing fast image acquisition. The two detectors are preceded by optical filters allowing the simultaneous acquisition of two separated channels: green channel (506–593 nm) and red channel (604–679 nm). The imaging overlay of the three channels and processing were performed by the operation software of the microscope. Additionally, a third GaAsP photomultiplier detector, connected to the microscope through an optical fiber and preceded by a dispersive element, was used to record the spectral profile of the TPEF signal (wavelength range 430 to 650 nm with a bandpass of 10 nm). The TPEF images and emission spectra of both porcine esophagus and microneedles were collected with excitation light at 850 or 1030 nm. Images were acquired with a typical field of view of 500 μm × 500 μm. To ensure consistency among images, detector gains were kept at the same relative values in all the images of the esophageal tissue. The focal plane was controlled by the motorized movement of the objective lens, enabling the imaging of specific regions at varying depths within the sample. Three-dimensional images were reconstructed from series of two-dimensional images (Z-stack) collected at predefined depths (Z-step). To prevent their structural deformation, microneedles were analyzed in the absence of water. Images of MNs applied to porcine tissue were obtained immediately after applying MN arrays to the luminal side of full-thickness esophagi.

## 4. Conclusions

The results obtained show that by applying soluble MNs, the transport of high-molecular-weight dextrans (70 and 150 kDa) across and into the mucosal tissue occurs very rapidly. A minimum length is required to cross the esophageal epithelium: 150 µm MNs did not produce any effect either in terms of amount permeated or tissue fluorescence, probably because they were too short to pierce the tissue (whose thickness is typically 400 µm). When 500 µm MNs were used, both types of dextrans permeated the tissue in similar amounts. The permeation was very quick, started within minutes, and leveled out in approx. 30 min, indicating that all available dextran was released. The use of solid MNs of the same length to pre-treat the tissue, followed by the application of a solution of the permeant, did not produce the same effect in terms of onset of permeation, which was found to be much slower. Increasing the length of MNs to 800 µm increased to a significant extent the amount permeated (*p* < 0.005 and *p* < 0.05, respectively, for FD-70 and FD-150; *t*-test), again in a similar manner for the two dextrans. The multiphoton microscopy analysis of MNs applied to the tissue allowed for the visualization in depth of the diffuse fluorescence signal from the partially dissolved MNs, confirming the presence of holes in the tissue and the permeation of the fluorescein-labeled dextrans. Since many other factors can influence skin permeability, further work will be required to optimize the MN array, for instance, by changing not only the length, but also the shape and pitch.

## Data Availability

The raw data supporting the conclusions of this article will be made available by the authors on request.

## References

[1] Rawas-Qalaji M., Thu H.E., Hussain Z. (2022). Oromucosal delivery of macromolecules: Challenges and recent developments to improve bioavailability. J. Control. Release.

[2] de la Torre B.G., Albericio F. (2024). The Pharmaceutical Industry in 2023: An Analysis of FDA Drug Approvals from the Perspective of Molecules. Molecules.

[3] Jena D., Srivastava N., Chauhan I., Verma M. (2024). Challenges and Therapeutic Approaches for the Protein Delivery System: A Review. Pharm. Nanotechnol..

[4] Nicolazzo J.A., Reed B.L., Finnin B.C. (2005). Buccal penetration enhancers—How do they really work?. J. Control. Release.

[5] Sohi H., Ahuja A., Ahmad F.J., Khar R.K. (2010). Critical evaluation of permeation enhancers for oral mucosal drug delivery. Drug Dev. Ind. Pharm..

[6] Padula C., Pescina S., Nicoli S., Santi P. (2018). New Insights on the Mechanism of Fatty Acids as Buccal Permeation Enhancers. Pharmaceutics.

[7] Fantini A., Giulio L., Delledonne A., Pescina S., Sissa C., Nicoli S., Santi P., Padula C. (2022). Buccal Permeation of Polysaccharide High Molecular Weight Compounds: Effect of Chemical Permeation Enhancers. Pharmaceutics.

[8] Ramadon D., McCrudden M.T.C., Courtenay A.J., Donnelly R.F. (2022). Enhancement strategies for transdermal drug delivery systems: Current trends and applications. Drug Deliv. Transl. Res..

[9] Gerstel M.S., Place V.A. (1976). Drug Delivery Device. U.S. Patent.

[10] Rzhevskiy A.S., Singh T.R.R., Donnelly R.F., Anissimov Y.G. (2018). Microneedles as the technique of drug delivery enhancement in diverse organs and tissues. J. Control. Release.

[11] Dsouza L., Ghate V.M., Lewis S.A. (2020). Derma rollers in therapy: The transition from cosmetics to transdermal drug delivery. Biomed. Microdevices.

[12] Waghule T., Singhvi G., Dubey S.K., Pandey M.M., Gupta G., Singh M., Dua K. (2019). Microneedles: A smart approach and increasing potential for transdermal drug delivery system. Biomed. Pharmacother..

[13] De Martino S., Battisti M., Napolitano F., Palladino A., Serpico L., Amendola E., Martone A., De Girolamo P., Squillace A., Dardano P. (2022). Effect of microneedles shape on skin penetration and transdermal drug administration. Biomater. Adv..

[14] Oliveira C., Teixeira J.A., Oliveira N., Ferreira S., Botelho C.M. (2024). Microneedles’ Device: Design, Fabrication, and Applications. Macromol.

[15] Le Z., Yu J., Quek Y.J., Bai B., Li X., Shou Y., Myint B., Xu C., Tay A. (2023). Design principles of microneedles for drug delivery and sampling applications. Mater. Today.

[16] Al-Qallaf B., Das D.B. (2009). Optimizing microneedle arrays for transdermal drug delivery: Extension to non-square distribution of microneedles. J. Drug Target..

[17] Ferreira L.E.N., Franz-Montan M., Benso B., Gill H.S. (2023). Microneedles for oral mucosal delivery—Current trends and perspective on future directions. Expert. Opin. Drug Deliv..

[18] Creighton R.L., Faber K.A., Tobos C.I., Doan M.A., Guo T., Woodrow K.A. (2024). Oral mucosal vaccination using integrated fiber microneedles. J. Control. Release.

[19] Caffarel-Salvador E., Kim S., Soares V., Tian R.Y., Stern S.R., Minahan D., Yona R., Lu X., Zakaria F.R., Collins J. (2021). A microneedle platform for buccal macromolecule delivery. Sci. Adv..

[20] Pireddu R., Schlich M., Marceddu S., Valenti D., Pini E., Fadda A.M., Lai F., Sinico C. (2020). Nanosuspensions and Microneedles Roller as a Combined Approach to Enhance Diclofenac Topical Bioavailability. Pharmaceutics.

[21] Choi J.J.E., Zwirner J., Ramani R.S., Ma S., Hussaini H.M., Waddell J.N., Hammer N. (2020). Mechanical properties of human oral mucosa tissues are site dependent: A combined biomechanical, histological and ultrastructural approach. Clin. Exp. Dent. Res..

[22] Farhat W., Chatelain F., Marret A., Faivre L., Arakelian L., Cattan P., Fuchs A. (2021). Trends in 3D bioprinting for esophageal tissue repair and reconstruction. Biomaterials.

[23] Manimaran R., Patel K.D., Lobo V.M., Kumbhar S.S., Venuganti V.V.K. (2023). Buccal mucosal application of dissolvable microneedle patch containing photosensitizer provides effective localized delivery and phototherapy against oral carcinoma. Int. J. Pharm..

[24] Diaz Del Consuelo I., Pizzolato G.P., Falson F., Guy R.H., Jacques Y. (2005). Evaluation of pig esophageal mucosa as a permeability barrier model for buccal tissue. J. Pharm. Sci..

[25] Verbaan F.J., Bal S.M., van den Berg D.J., Groenink W.H., Verpoorten H., Lüttge R., Bouwstra J.A. (2007). Assembled microneedle arrays enhance the transport of compounds varying over a large range of molecular weight across human dermatomed skin. J. Control. Release.

[26] Bisgaard S.I., Nguyen L.Q., Bøgh K.L., Keller S.S. (2023). Dermal tissue penetration of in-plane silicon microneedles evaluated in skin-simulating hydrogel, rat skin and porcine skin. Biomater. Adv..

[27] Matsukawa Y., Lee V.H., Crandall E.D., Kim K.J. (1997). Size-dependent dextran transport across rat alveolar epithelial cell monolayers. J. Pharm. Sci..

[28] Ambati J., Canakis C.S., Miller J.W., Gragoudas E.S., Edwards A., Weissgold D.J., Kim I., Delori F.C., Adamis A.P. (2000). Diffusion of high molecular weight compounds through sclera. Investig. Ophthalmol. Vis. Sci..

[29] Hutton A.R.J., McCrudden M.T.C., Larrañeta E., Donnelly R.F. (2020). Influence of molecular weight on transdermal delivery of model macromolecules using hydrogel-forming microneedles: Potential to enhance the administration of novel low molecular weight biotherapeutics. J. Mater. Chem. B.

[30] Diaz-Del Consuelo I., Jacques Y., Pizzolato G.P., Guy R.H., Falson F. (2005). Comparison of the lipid composition of porcine buccal and esophageal permeability barriers. Arch. Oral Biol..

[31] Casula L., Pireddu R., Cardia M.C., Pini E., Valenti D., Schlich M., Sinico C., Marceddu S., Dragićević N., Fadda A.M. (2023). Nanosuspension-Based Dissolvable Microneedle Arrays to Enhance Diclofenac Skin Delivery. Pharmaceutics.

